# Preliminary Investigation on Micro-Needling with Low-Level LED Therapy and Growth Factors in Hair Loss Related to COVID-19

**DOI:** 10.3390/jcm11195760

**Published:** 2022-09-28

**Authors:** Pietro Gentile

**Affiliations:** 1 Plastic and Reconstructive Surgery, Department of Surgical Science, University of “Tor Vergata”, 00133 Rome, Italy; pietrogentile2004@libero.it; Tel.: +39-3388-5154-79; 2 Academy of International Regenerative Medicine & Surgery Societies (AIRMESS), 1201 Geneva, Switzerland

**Keywords:** COVID-19 hair loss, COVID-19 hair regrowth, SARS-CoV-2 hair, regenerative plastic surgery, plastic surgery

## Abstract

The incidence of hair loss (HL) and telogen effluvium (TE) has increased due to the spread of the coronavirus disease (COVID-19) induced by severe acute respiratory syndrome coronavirus 2 (SARS-CoV-2). New biotechnologies based on micro-needling (MND) with Low-Level LED Therapy (LLLT) and Growth Factors (GFs) used for hair re-growth (HR-G) in Androgenetic Alopecia (AGA) need to be standardized also in HL and TE related to COVID-19. This article aims to describe the preliminary clinical results obtained from an open-label case-series observational study. MND with LLLT and GFs was used in patients affected by HL and TE-related to COVID-19. In total, 10 patients (6 men were identified in stage I–III vertex according to the Norwood–Hamilton scale, and 4 women were identified in stage I–II according to the Ludwig scale) were enrolled and analyzed after screening (exclusion and inclusion criteria evaluation). HR-G assessment was analyzed through photography, physician’s and patient’s global assessment scale, and standardized phototrichograms during a short follow-up: T0—baseline; T1—20 weeks (wks). In the targeted area computerized trichograms showed encouraging results with a hair density increase of 11 ± 2 hairs/cm^2^ at T1 after 20 wks (20 wks vs. 0 wks) compared with baseline (58 ± 2 hairs/cm^2^ at T1 versus 47 ± 2 hairs/cm^2^ at baseline) with a not quite statistically significant difference in HR-G (*p* = 0.0690). The preliminary effectiveness of MND with LLLT and GFs use has been demonstrated in mild-to-moderate HL and TE related to COVID-19. Further controlled trials are required to confirm these preliminary results.

## 1. Introduction

Hair loss (HL) can be classified into several types including androgenetic alopecia (AGA), alopecia areata (AA), and telogen effluvium (TE). AGA is the most frequent HL cause affecting a mean of 80% of white males and 40% of females, determining a male pattern hair loss (MPHL) and a female pattern hair loss (FPHL) [1,2,3,4]. As known, the cause of AGA is a combination of genetics and male hormones, while the cause of AA is autoimmune, and the cause of TE is typically a physically or psychologically stressful event [1,2,3,4]. Starting in 2021, an evolving body of literature has been associated with HL disorders related to COVID-19. A simultaneous rise in public apprehension about HL and the rising number of COVID-19 cases indicates a connection. It is expected that the pathogenetic components of COVID-19’s psychological problems will either cause or aggravate HL. Scientific research in the HL field exists even to develop a non-invasive biotechnology to increase hair re-growth (HR-G) in patients suffering from AGA, AA, and TE. The number of papers analyzing the effectiveness of Platelet-Rich Plasma (PRP) and Growth Factors (GFs) [1,2,3,4,5], Human Follicle Mesenchymal Stem Cells (HF-MSCs) [6,7], Micro-needling (MND) [8,9], and Low-Level LED Therapy (LLLT) [10] in HL disorders has exponentially increased during the last 10 years. Consolidated effectiveness of PRP use in patients suffering from AGA has been demonstrated [1,2,3,4,5] as the positive impact of HF-MSCs [6,7] while more recently the combined use of the MND technique with LLLT aiming to improve HR-G has been described [8,9,10].

SARS-CoV-2 can increase plasma levels of important proinflammatory cytokines such as IL-1, IL-6, IL-2, IL-17, interferon (IFN-), monocyte chemoattractant protein 1 (MCP-1), IP-10, and many others. This can result in both local and systemic inflammation in COVID-19 patients (acute and convalescent) [11]. It is conceivable that such a sudden increase in the levels of numerous cytokines in the blood in COVID-19 patients exposes follicular cells to potently inhibiting and disrupting forces [11,12,13,14,15,16]. As a result, the cycle of hair growth is disturbed, and the PHL process is considerably accelerated [11,12,13,14,15,16]. This mechanism may account for the rapid onset of HL following SARS-CoV-2 infection. Consequently, oxidative stress and inflammation appear to be fundamental in the trigger of COVID-19-related HL and TE [11,12,13,14,15,16].

Despite the increasing incidence of HL disorders in COVID-19 patients, no papers based on MND, LLLT, and GFs were found during the literature review.

This preliminary work aims to describe the encouraging impact of LLLT with MND and GFs on HR-G in patients suffering from HL and/or TE related to COVID-19.

## 2. Methods

### 2.1. Inclusion and Exclusion Criteria

Inclusion criteria were age 18–70 years, MPHL from stage I to stage III vertex according to the Norwood–Hamilton classification, FPHL from stage I to stage II according to the Ludwig scale, and a positive history of COVID-19 during the anamnesis.

There were systemic and local exclusion criteria. The use of anticoagulants, chronic dermatologic conditions such as eczema, psoriasis, or scalp infections, a history of slow wound healing or keloid formation, a history of thyroid dysfunction and/or autoimmune disorders, use of Finasteride^®^ or analogous medications, and/or anti-androgens within the previous year were among the systemic exclusion criteria. An MPHL, over III vertex degree, and an FPHL, over II degree, as well as the usage of lotions such as Minoxidil^®^, prostaglandin analogs, retinoids, or corticosteroids during the previous year, were local exclusion criteria.

### 2.2. Study Overview

A preliminary open-label case-series observational clinical investigation was carried out respecting the rules set forth in the Declaration of Helsinki and internationally consented ethics in clinical research [17]. A high-quality assessment was performed following the Strengthening the Reporting of Observational Studies in Epidemiology (STROBE) checklist [18]. All participants signed the informed consent before any treatment, reporting information on applicative protocol, benefits, side effects, and alternative procedure. The field of the current investigation was the object of a research project called “Evaluation of the potential use of regenerative strategies in the treatment of diseases associated with COVID-19” approved on March 15, 2022, by the University “Tor Vergata”, Rome, Italy, and supported with Unique Project Code (CUP): E83C22001960005.

### 2.3. Endpoint Definition

Information on patients (age, sex, race, hair-loss degree), interventions (targeted area), and follow-up (20 weeks—wks) was collected. Data on hair density (HD), hair count (HC), session frequency (days/week), treatment duration (wks), and patients’ clinical results were analyzed.

The differences in HD between the baseline (T0), and LLLT with MND and GF treatments at 20 wks (T1), evaluated with instrumental trichoscopy, were the primary outcomes. The *p*-value indicated LLLT with MND and GFs as being an effective treatment option when compared to the baseline.

Clinical results were analyzed subjectively and objectively for analysis. While the recruited participants completed the “subjective evaluation”, the principal investigator (P.G.) completed the “objective evaluation”. The author’s analysis was based on a clinical, comprehensive picture analysis using a six-point scale (1—excellent; 2—good; 3—discreet; 4—enough; 5—poor; 6—inadequate). The same six degrees that were previously described were used for the patient self-analysis. Itching, a little redness, a little numbness of the treated portion, and headaches were additional characteristics or variables taken into account during the outcomes analysis.

### 2.4. Micro-Needling (MND) Combined with Low-Level Light Therapy (LLLT) and Growth Factors (GFs): Protocol and Device

**Figure 1:**
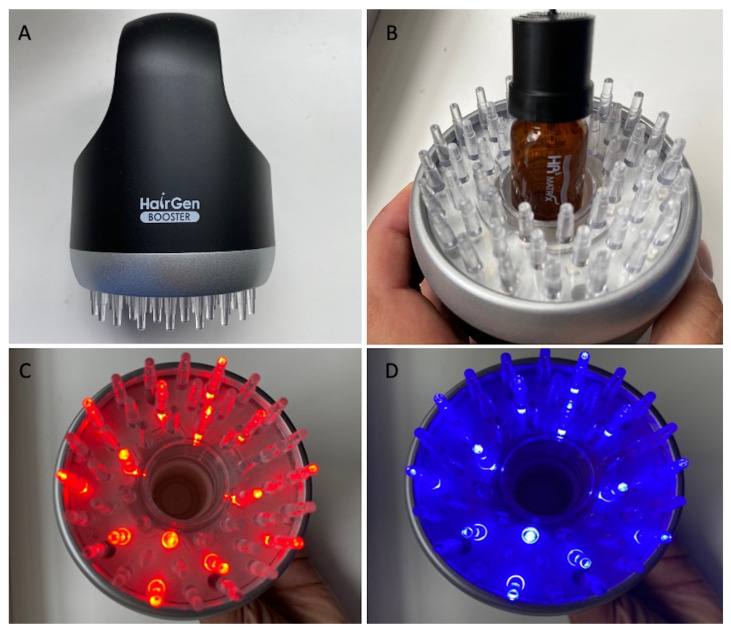
MND and LLLT protocol (**A**) Hairgen Booster^®^ (DTS MG Co., Ltd., Seoul, Korea, #B108-147); (**B**) Hairgen Booster^®^ during the insertion of HR3 Matrix Hair Solution Alpha^®^ (DTS MG Co., Ltd., Seoul, Korea, #B108-147) connected with an HR3 Matrix Hair Stamp^®^ (DTS MG Co., Ltd., Seoul, Korea, #B108-147); (**C**) red light emission (wavelength 640 nm); (**D**) blue light emission (wavelength 423 nm).

MND with LLLT and GFs protocol was based on two treatments weekly for 5 months using at home the device Hairgen Booster^®^ (DTS MG Co., Ltd., Seoul, Korea, #B108-147) (Figure 1A) permitting the injection of GFs contained in a vial of hair solution (HR3 Matrix Hair Solution Alpha^®^) through a micro-needling stamp (Figure 1B). The participation period for every enrolled subject was five months. The whole treatment course included 40 sessions conducted twice per week for 20 wks (T1). Patients were re-evaluated at five months (T1—20 wks) (five months after the first application). The Hairgen Booster^®^ once applied and passed on the targeted area, maintains the same distance between the scalp and the led, permitting contextually, the sterile infiltration (0.22 μm) by MND stamp of a solution (HR3 Matrix Hair Solution Alpha^®^—Repilosome-EPH1) containing human growth hormone (GH), Epidermal Growth Factors (EGFs), Vasoactive Intestinal Peptide (VIP), and several polypeptides (sh-Polypeptide-7, sh-Oligopeptide-1, sh-Polypeptide-71), glycerin, lecithin, polysorbate 60, sodium citrate, citric acid, phenoxyethanol, and water (Figure 1B). The accuracy of the procedure, however, depended on the use of one’s hands and on how the device was passed on the scalp. Hairgen Booster^®^ emits red light (Figure 1C), with a wavelength of 640 nm, improving cell metabolism, blood circulation, and nutrition supply to capillaries, and blue light (Figure 1D) with a wavelength of 423 nm activating the keratin present in the hair shaft, and diminishing the sebaceous gland and the grease of the scalp.

### 2.5. Phototrichograms Collection

Phototrichograms (Figure 5A,B) were gathered from all scalps by a trained evaluator (a dermatologist not involved in the study) using Fotofinder video-epiluminescence microscopy (FotoFinder Systems; http://www.fotofinder.de (accessed on 11 September 2022)) combined with the Trichoscan digital image evaluation (Tricholog GmbH and Datinf GmbH; http://trichoscan.com (accessed on 11 September 2022)). In all participants, in both the treatment and control half-heads, two targeted areas (TAs) of HL were identified for the subsequent trichogram.

### 2.6. Statistical Analysis

HD was expressed as the mean ± the standard deviation (SD). One-way repeated measures analysis of variance was used to compare HD between the various time points, and the Sidak test was used for post hoc analysis. All tests were two-tailed, and a value of *p* < 0.05 was considered statistically significant. All analyses were performed using an online *p*-value calculator (https://www.graphpad.com/quickcalcs/ttest1.cfm (accessed on 11 September 2022)).

## 3. Results

### 3.1. Patient Assessment

**Figure 2:**
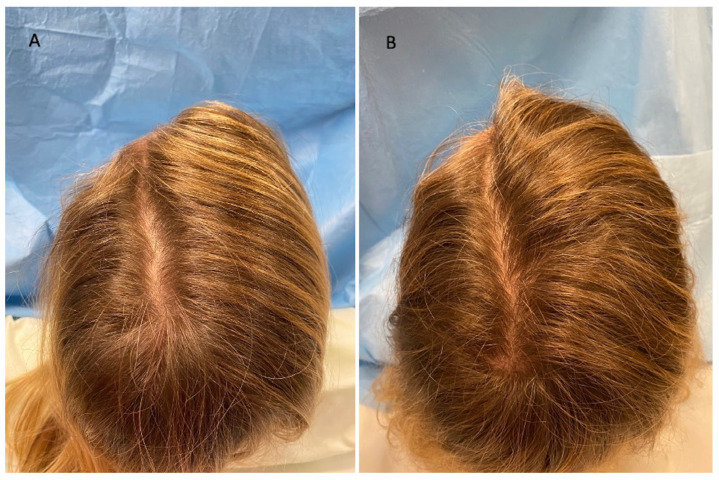
Caucasian female patient treated with LLLT, MND, and GFs protocol. (**A**) Pre-operative view of the scalp of a 41 year-old female patient affected by AGA of II degree according to the Ludwig scale and several episodes of TE during the pandemic period and in particular 1 month after the COVID-19 positivity, with hair loss localized in the frontal, temporal, and parietal areas; (**B**) post-operative view at T1 (20 wks) after treatment with detail of HR-G in the parietal area.

**Figure 3:**
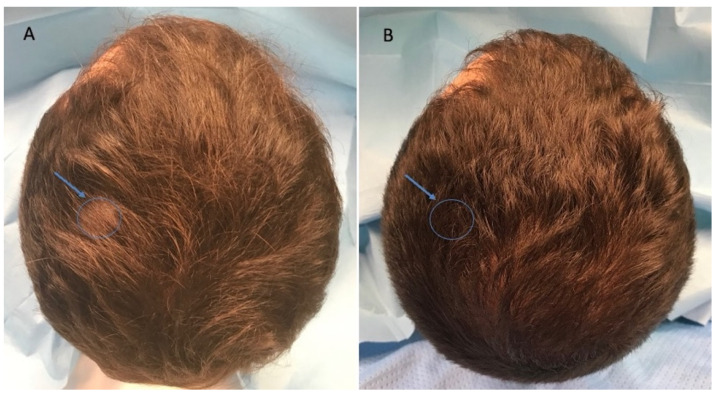
Caucasian male patient treated with LLLT, MND, and GFs protocol. (**A**) Pre-operative view of the scalp of a 32 year-old male patient affected by AGA of II degree according to the Norwood scale and several episodes of TE during the pandemic period and in particular 3 months after the COVID-19 positivity, with hair loss localized in the frontal, temporal, and parietal areas; the blue arrow and related circle identifies the targeted area (TA) in which the Trichoscan evaluation was performed. (**B**) Post-operative view at T1 (20 wks) after treatment with detail of HR-G in the left parietal area (arrow and related circle).

**Figure 4:**
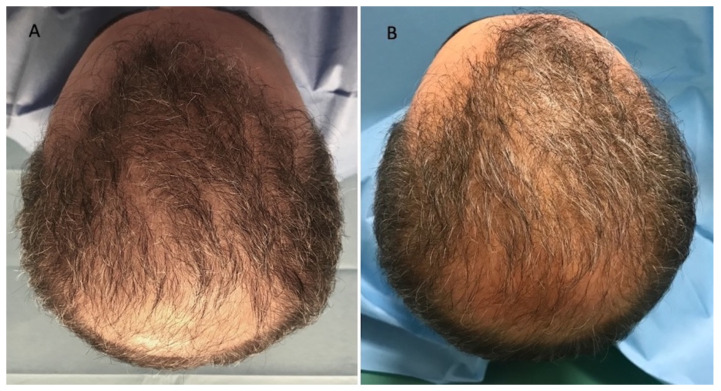
Caucasian male patient treated with LLLT, MND, and GFs protocol. (**A**) Pre-operative view of the scalp of a 68 year-old male patient affected by AGA of III vertex degree according to the Norwood scale, with hair loss localized in the frontal, temporal, parietal, and vertex areas; (**B**) post-operative view at T1 (20 wks) after treatment with detail of HR-G in the treated areas (frontal, parietal, and vertex).

This case series was performed on 10 patients, treated since September 2020, aged 23–68 years, of which 6 men were identified in stage I–III vertex according to the Norwood–Hamilton scale (Figure 3A and Figure 4A), and 4 women were identified in stage I–II according to the Ludwig scale (Figure 2A). All the patients presented a positive history of COVID-19 during the anamnesis. In detail, the diagnosis of severe acute respiratory syndrome coronavirus 2 (SARS-CoV-2) infection was performed via a nose swab polymerase chain reaction (PCR) test. A positive test for SARS-CoV-2 was revealed in a period of 1–6 months before the hair loss onset in all patients analyzed. Additionally, all the patients treated affirmed to have received the vaccination anti-COVID-19 with one dose (1 male and 1 female), two doses (2 males and 2 females), and three doses (3 males and 1 female). In Table 1, patient characteristics are listed. During a follow-up, the HR-G was evaluated using photography, the patient’s and doctor’s global assessment scales, and standardized phototrichograms: T0—baseline; T1—20 wks (Figure 2B, Figure 3B and Figure 4B).

### 3.2. Trichoscopy Analysis

**Figure 5:**
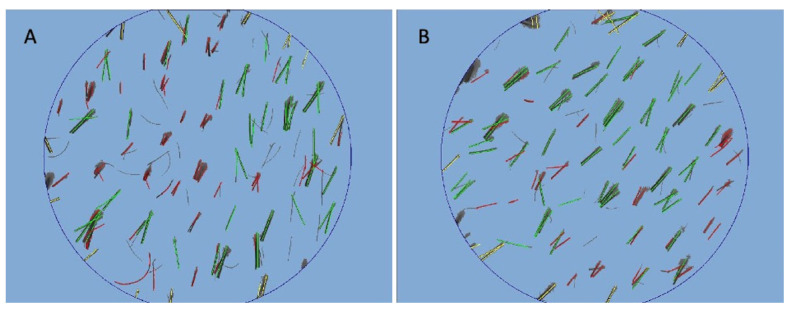
Trichoscan digital image analysis performed by Fotofinder in a patient is shown in Figure 2. (**A**) At T0 pre-operative hair density was 61 ± 2 hairs/cm^2^ and proportions of telogen and anagen hairs were 34.4% and 52.3%, respectively; (**B**) at T1 (20 wks) post-operative hair density was 72 ± 2 hairs/cm^2^, and proportions of telogen and anagen hairs were 40.1% and 49.9%, respectively.

Encouraging results were represented by an HD increase of 11 ± 2 hairs/cm^2^ at T1 after 20 wks (20 wks vs. 0 wks) in the TA compared with baseline (58 ± 2 hairs/cm^2^ at T1 versus 47 ± 2 hairs/cm^2^ at baseline) using trichogram analysis (Figure 5B), with a not quite statistically significant difference in HR-G (*p* = 0.0690). The control area (CA) displayed a mean decrease of 4.3 hairs/cm^2^ (control vs. treatment: *p* < 0.0001). All the details in terms of HD are reported in Table 2. No statistically significant differences in vellus HD among the baseline and T1 were observed.

### 3.3. Clinical Evaluation

Regarding the investigator evaluation, scores ranged from 2 to 5 (*p* = 0.135), and 6 patients (60%) (4 males, 2 females) that underwent the LLLT, MND, and GFs, reported good results about global scalp coverage and hair thickness (Figure 2B) versus 4 patients (40%) (2 males, 2 females) with ineffective results.

Regarding the patient evaluations, scores ranged from 1 to 4 (*p* = 0.044), and 7 patients (70%) (5 males, 2 females) reported a good satisfaction about global scalp coverage versus only 3 patients (30%) (1 male, 2 females) who reported ineffective results.

The results reported showed the men enrolled in the study to be more satisfied than the women. The analysis of the satisfaction grade assessment questionnaire revealed that all respondents were sufficiently informed about the protocol, benefits, and side effects (including the ineffective results and risk of the high possibility to repeat the procedure more times) of Hairgen Booster^®^ and would opt to undertake hair bio-stimulation.

### 3.4. Limitations

The most important limitations were both the small cohort of subjects enrolled represented by only 10 patients and the evidence-based medicine (EBM) level 4 study, represented by a “case series” analysis without a control group. Furthermore, the “open-label” study, as opposed to “single-blinded” or “double-blinded”, inhibits having an entirely impartial evaluation, or that in any case was not impacted in any way by knowledge of having received one therapy rather than another. This entails a bias in the research.

## 4. Discussion

Clinical studies carried out in Spain [13,14] and India [16] revealed that hospitalized COVID-19 patients had greater rates and more severe PHL than age-matched, non-infected populations. The first preliminary inquiry into it involved a descriptive study on 41 Caucasian males who were diagnosed with bilateral SARS-CoV-2 pneumonia and were admitted to hospitals in Spain (mean age = 58 years). In total, 39% of patients with considerable MPHL, who made up 71% of the patients, had a severe involvement [13]. Those preliminary findings were corroborated by a subsequent multicenter study, which found that 42% (95% CI: 29–55%) of women and 79% (95% CI: 70–85%) of males had significant PHL. These results are at odds with the anticipated prevalence rates in people with similar ages and races. In a comparable white population, MPHL prevalence is predicted to be between 31 and 53%, and FPHL prevalence to be at most 38% [14,15]. As a result, the data so far indicate that hospitalized COVID-19 patients had a much higher prevalence and severity of PHL. Notably, those with more advanced HL experienced poorer clinical outcomes (use of ventilators and deaths). Diffuse alopecia seems to be a prominent COVID-19 aftereffect, in addition to having a good connection with the SARS-CoV-2 infection. In order to determine the prevalence and determinants of COVID-19 clinical sequelae, a significant longitudinal study with 538 COVID-19 survivors and 184 controls was conducted in Wuhan, China [19]. Alopecia was among the most common complaints in convalescent COVID-19 patients three to four months after discharge, with women reporting it more frequently. After contracting SARS-CoV-2, over half of the female participants started losing their hair, in contrast to the control group, which had no such cases. A total of 73% of affected people initially noticed baldness after being discharged, while 27% developed it while they were hospitalized [19]. At least a portion of the new-onset alopecia cases in this study are suspected to have premature or worsened FPHL due to the timing of symptoms. As a potential explanation for the link between PHL and COVID-19, systemic inflammation may play a significant role as a common underlying disease. This significant factor may also support Wambier et al. [16] findings that patients with severe COVID-19 experience higher degrees of hair loss. Another putative pathogenetic mechanism that links SARS-CoV-2-related lung injury with impaired hair growth is hypoxia, which can result in skin ischemia. Ex vivo and in vivo tests by Kato et al. [20] showed that anagen hairs exposed to ischemia experienced significant decreases in hair-growth rate, hair-shaft size, and color. Hair growth and hair cycling are negatively impacted by hypoxia in COVID-19 patients, which could justify the therapeutic use of regenerative treatments such MND, LLLT, and GFs that have protective effects against ischemia injury. All these treatments are intended to increase scalp angiogenesis. It is believed that encouraging angiogenesis and preventing ischemia in the cells are crucial treatment strategies for COVID-19-induced hair loss.

In the present study, at 20 wks, an HD increase of 23.5% (58 ± 2 hairs/cm^2^ at T1 versus 47 ± 2 hairs/cm^2^ at baseline, with 11 ± 2 hairs/cm^2^ for HD increase) was observed using trichogram analysis.

Currently, clinical data regarding HD evaluation, using regenerative strategies, are not yet published in patients suffering from HL and TE strictly related to COVID-19, but only in patients suffering from AGA or PHL.

Previous studies performed by the author Gentile P et al. [6] reported an HD increase of 28% and 29% at 23 wks using PRP and micrografts containing human follicle stem cells, respectively, in patients suffering from AGA. The mean change in HD from baseline to week 24 in Suchonwanit’s study [21] using LLLT was 10.21 ± 3.25 hairs/cm^2^ in the LLLT group against 3.95 ± 1.32 hairs/cm^2^ in the sham group. These data appear to align with the previously published data by Gentile et al. [9] (12 ± 2 hairs/cm^2^ at 16 wks) using MND and LLT in AGA patients.

## 5. Conclusions

In conclusion, this preliminary case-series “open-label” observational study analyzed the potential role of MND, LLLT, and GFs in HL related to COVID-19. The reported results show promise and suggest that using these regenerative strategies to treat COVID-19-related HL and TE could prove effective. They also found men to be more satisfied with the treatment outcomes than women. Further research via randomized and controlled investigations is needed to define standardized protocols, and large-scale regenerative therapy trials still need to be conducted to confirm their effectiveness.

## Figures and Tables

**Table 1:** Patient characteristics.

Patients	Gender	Hamilton–Norwood Degree	Ludwig Degree	Targeted Area	Age	Race	Doses	Vaccine
1	Male	III-vertex	-	Frontal, temporal, parietal, vertex	23	Caucasian	1 dose	Pfizer
2	Male	III-vertex	-	Frontal, temporal, parietal, vertex	43	Caucasian	2 doses	Pfizer
3	Male	III-vertex	-	Frontal, temporal, parietal, vertex	68	Caucasian	3 doses	Pfizer
4	Male	IIa	-	Frontal, temporal, parietal,	45	Caucasian	3 doses	Pfizer
5	Male	II	-	Frontal, temporal, parietal	32	Caucasian	2 doses	Pfizer
6	Male	I	-	Frontal, parietal	38	Caucasian	3 doses	Pfizer
7	Female	-	II	Frontal, temporal, parietal, vertex	41	Caucasian	2 doses	Pfizer
8	Female	-	II	Frontal, temporal, parietal, vertex	46	Caucasian	2 doses	Pfizer
9	Female	-	II	Frontal, temporal, parietal, vertex	54	Caucasian	3 doses	Pfizer
10	Female	-	I	Frontal, parietal	31	Caucasian	1 dose	Pfizer

**Table 2:** In vivo evaluation using trichoscopy analysis in terms of hair density (HD) (hairs/cm^2^) improvement.

Patients	Procedure	Hair Density (T0)	Hair Density (T1—16 wks)
1	DTSMG MTS stamp + HR3 matrix	34 ± 2 hairs/cm^2^	45 ± 2 hairs/cm^2^
2	DTSMG MTS stamp + HR3 matrix	45 ± 2 hairs/cm^2^	56 ± 2 hairs/cm^2^
3	DTSMG MTS stamp + HR3 matrix	61 ± 2 hairs/cm^2^	72 ± 2 hairs/cm^2^
4	DTSMG MTS stamp + HR3 matrix	30 ± 2 hairs/cm^2^	41 ± 2 hairs/cm^2^
5	DTSMG MTS stamp + HR3 matrix	65 ± 2 hairs/cm^2^	76 ± 2 hairs/cm^2^
6	DTSMG MTS stamp + HR3 matrix	33 ± 2 hairs/cm^2^	44 ± 2 hairs/cm^2^
7	DTSMG MTS stamp + HR3 matrix	63 ± 2 hairs/cm^2^	74 ± 2 hairs/cm^2^
8	DTSMG MTS stamp + HR3 matrix	48 ± 2 hairs/cm^2^	59 ± 2 hairs/cm^2^
9	DTSMG MTS stamp + HR3 matrix	45 ± 2 hairs/cm^2^	56 ± 2 hairs/cm^2^
10	DTSMG MTS stamp + HR3 matrix	39 ± 2 hairs/cm^2^	50 ± 2 hairs/cm^2^
